# Effects of Various Annealing on the Thermoluminescence Behavior of Hexagonal Boron Nitride: A Group III–Nitride Semiconductor

**DOI:** 10.1002/bio.70298

**Published:** 2025-08-31

**Authors:** Muhammed Hatib, Huseyin Toktamis

**Affiliations:** ^1^ Department of Engineering Physics University of Gaziantep Gaziantep Turkey

**Keywords:** annealing, Group III–nitride semiconductor, hexagonal boron nitride (h‐BN), radiation dosimeters, thermoluminescence, wide bandgap semiconductor

## Abstract

This study investigated the effects of different annealing methods on the dosimetric properties of the Group III–nitride semiconductor hexagonal boron nitride (h‐BN) to get the optimal annealing corresponding to an ideal thermoluminescence (TL) glow curve. Two distinct annealing methods were applied to 15 powder samples. The first technique involved modulating the temperature within the range of 200°C to 1000°C while keeping the duration fixed at 30 min. In contrast, the second approach varied the annealing duration between 1 and 120 min while maintaining a constant temperature of 900°C. The resultant TL glow curves displayed two distinct dosimetric peaks at 160°C and 255°C for annealing temperatures between 600°C and 900°C and durations ranging from 1 to 30 min. Nevertheless, at elevated annealing temperatures and extended durations, an additional shallow peak was identified at approximately 85°C. A comparative evaluation of the findings revealed that optimal TL performance is attained at 900°C for 30 min, supplemented by an additional storage period of 10 min to mitigate the impact of the shallow peak. Moreover, SEM and XRD analysis showed a more stable and homogeneous microstructure under this process.

## Graphical abstract

With this study, the effects of different annealing methods on the dosimetric properties of the Group III–nitride semiconductor hexagonal boron nitride (h‐BN) were investigated. The TL glow curves displayed two peaks at 160°C and 255°C for annealing temperatures between 600°C and 900°C and durations of 1–30 min. Optimal TL performance is attained at 900°C for 30 min, with an additional storage period of 10 min to mitigate the shallow peak.

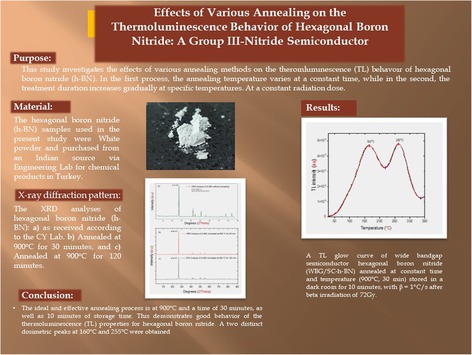

## 1 Introduction

Beyond its confirmed role in improving crystallinity, activating dopants, and repairing defects, annealing is considered an effective technique to induce the optical and electronic properties of insulating materials and wide bandgap semiconductors, such as hexagonal boron nitride (h‐BN), a member of Group III–nitrides [1, 2, 3]. This treatment plays an essential role not only in modifying the energy bandgap but also in inducing significant changes in charge carrier dynamics [4].

**FIGURE 1:**
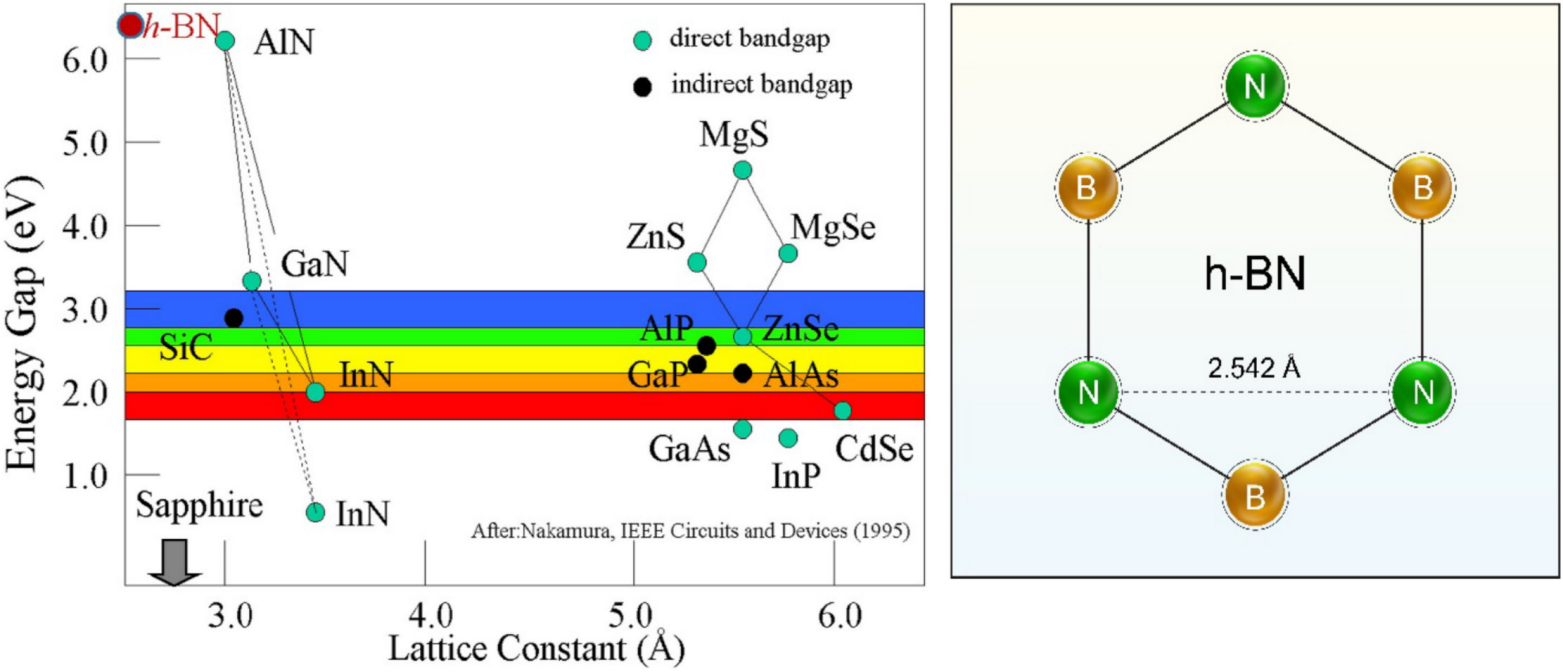
a) Energy bandgap vs. *a*‐lattice constant for common compound semiconductors including Group III–nitrides [13]. Credit: Reprinted with permission from Elsevier. b) The crystal 2D structure of hexagonal boron nitride (h‐BN).

Due to its distinct and respective turn in enhancing the efficiency of electronic devices, understanding the effects on optical properties, such as thermoluminescence (TL) and photoluminescence (PL), electroluminescence represents a right step toward the empowerment of this performance and the development of innovative optical applications. This will further establish the prominent role of wide‐bandgap semiconductors in next‐generation semiconductor technologies [5, 6, 7]. Amid increasing economic inflation and rising costs of electronic devices and applications, there has been a raised necessity to conduct more research and develop high‐efficiency electronic devices and applications capable of withstanding extreme usage conditions [8]. Hexagonal boron nitride is widely available and cost‐effective, making it a practical choice for high‐power electronics and optoelectronics. It demonstrates high thermal and chemical stability, along with exceptional insulating properties, which contribute to its resilience at elevated temperatures [9, 10]. These characteristics, along with high thermal conductivity and electronic mobility, allow it to be used as a substrate for the growth of other materials, such as graphene [10, 11]. Meanwhile, the optical properties, including a large energy gap (~6.5 eV) and a small crystal lattice [10, 12, 13], as shown in Figure 1, make it a good candidate for UV emitters and detectors used in energy‐efficient lighting and sensing applications [14, 15, 16], as well as its utilization in cosmetics as a texture enhancer for creams and powders' spreadability and blendability [17].

In this study, the TL behavior of the Group III–nitride semiconductor h‐BN was investigated under various annealing processes. In the first phase, the annealing temperature varies at a constant time, while in the second phase, the treatment duration increases gradually at specific temperatures. Unlike most studies that focus solely on temperature variation, this research also explores the effect of annealing duration to get a more profound vision of the influence of heat treatment. A constant radiation dose was applied at each thermal treatment stage, followed by the plotting of TL glow curves. Finally, a comparison of the resultant plots will conclude the annealing process that aligns with the optimal TL glow curve, according to TL theory [18, 19].

**FIGURE 2:**
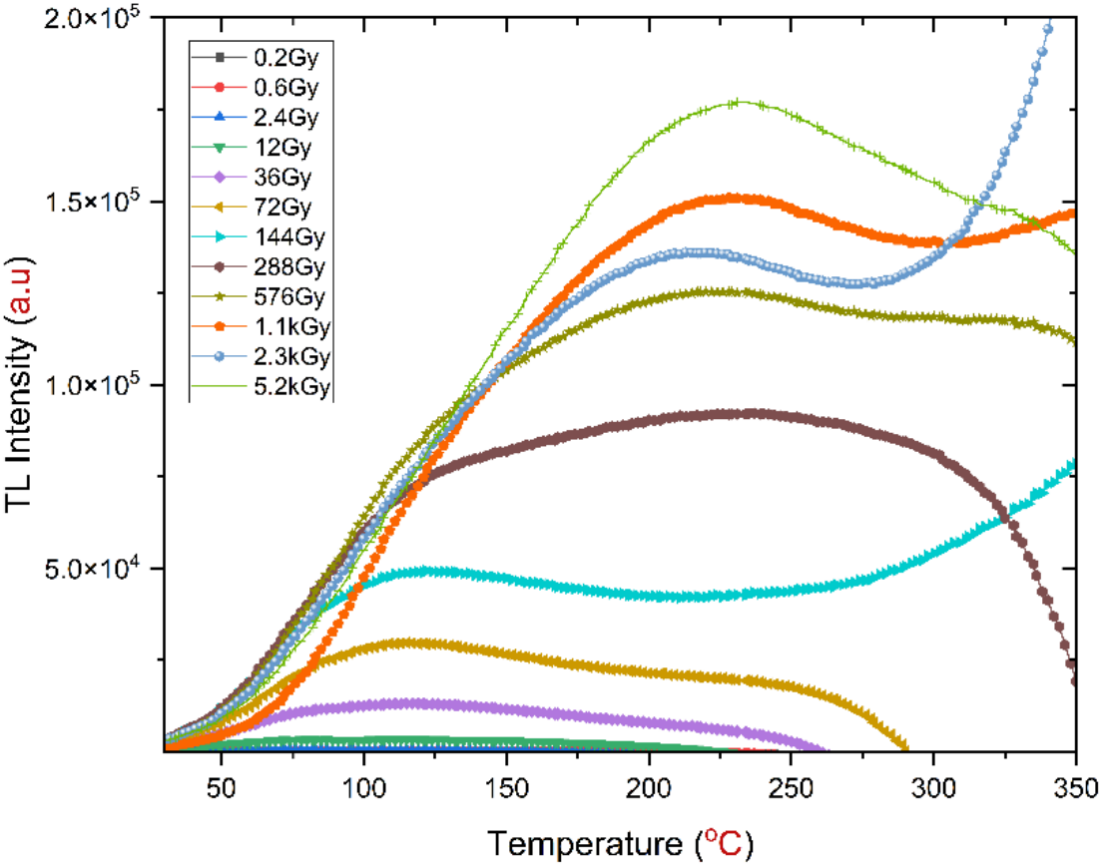
TL glow curves for non‐annealed h‐BN at various radiation dose levels (0.2 Gy to 5.2 kGy) with a heating rate of 1°C/s.

However, an initial study was conducted on a 25 mg h‐BN powder sample without thermal treatment, where progressively increasing radiation doses were applied. The results showed broad, continuous TL glow curves, as illustrated in Figure 2. This means overlapping, deep, and adjacent traps. That was not useful for studying TL properties according to the theoretical framework [18, 19]. Ideally, TL behavior is characterized by sharp, symmetrical, and distinct peaks, each corresponding to a specific trap depth, through which one can study the optical properties of.

The analysis of this study's findings may encourage further research on the significance of thermal treatment in exploring the optical properties of wide bandgap semiconductors, including Group III nitrides, as studies in this field remain insufficient.

## 2 Experiment

### 2.1 Material

**FIGURE 3:**
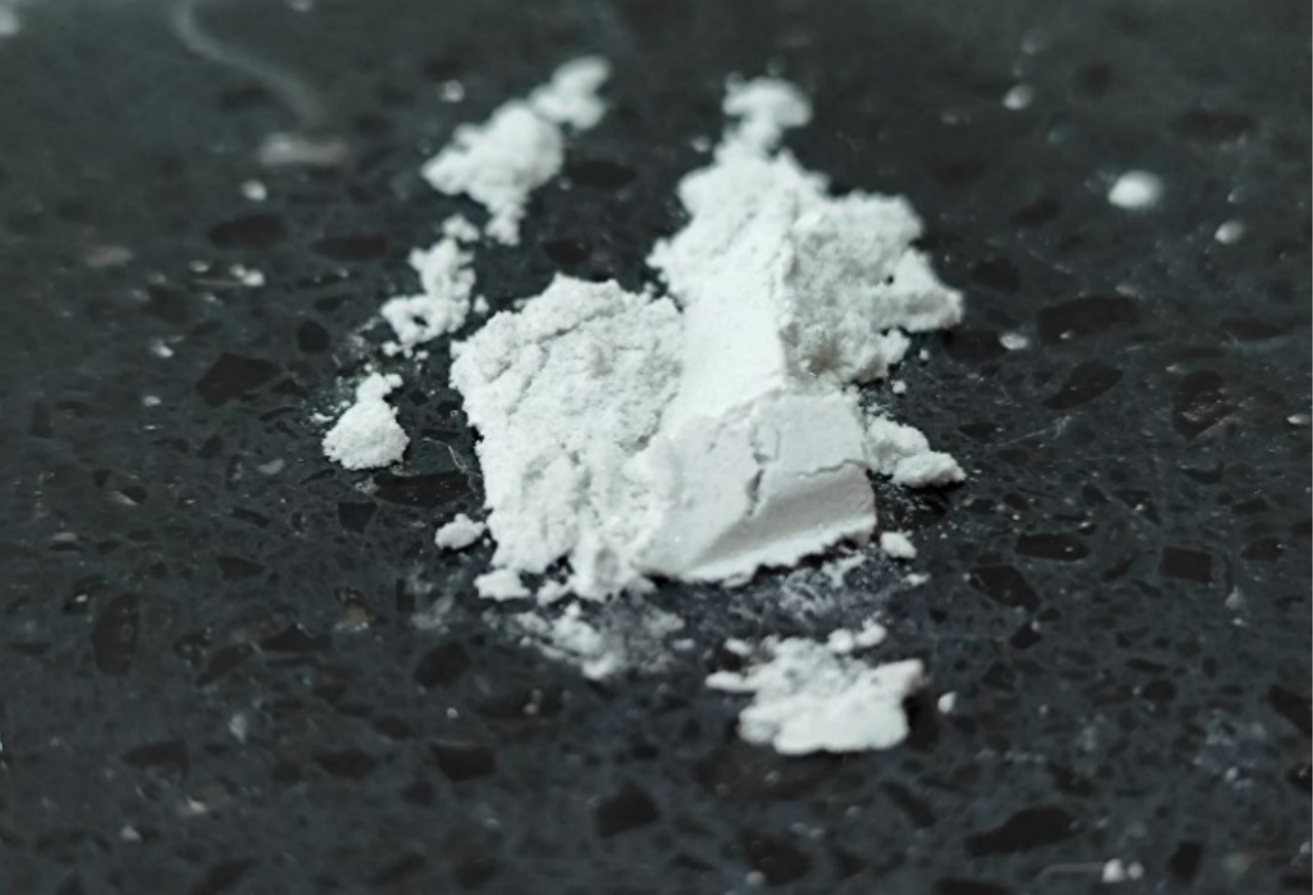
h‐BN sample used in this study.

The h‐BN samples used in the present study were powder and purchased from an Indian source via CY Engineering Lab for chemical products in Turkey. It is white boron nitride, as seen in Figure 3.

Table 1 shows the chemical structure of hexagonal boron nitride obtained from Cy Engineering (XRF).

**TABLE 1:** Chemical structure of hexagonal boron nitride from CY Engineering.

Formula	h‐BN	CaO	SiO_2_	B_2_O_3_
Percentage	>99.8	<0.2	<0.05	<0.1

### 2.2 Equipment

Samples were weighed up to 25 mg using a high‐precision digital balance and annealed in a high‐temperature furnace at temperatures ranging from 200°C to 1000°C for varying durations between 1 and 120 min. The annealing process was conducted in platinum crucibles to ensure chemical stability and prevent contamination. Following annealing, each sample was exposed to a calibrated ^90^Sr–^90^Y beta radiation (β‐rays) source at room temperature. The radioactive source activity is 100 mCi, delivering a radiation dose rate of 2.64 Gy/min (0.0438 Gy/s), integrated with a 9010 optical dating system. All irradiated samples were read out by a Harshaw QS 3500 manual TLD reader at a 1°C/s heating rate. A standard colorless glass optical filter was positioned between the planchette and the photomultiplier tube within the TLD reader to mitigate interference from infrared radiation emitted by both the heating element and the sample.

## 3 Experimental Procedures

**FIGURE 4:**
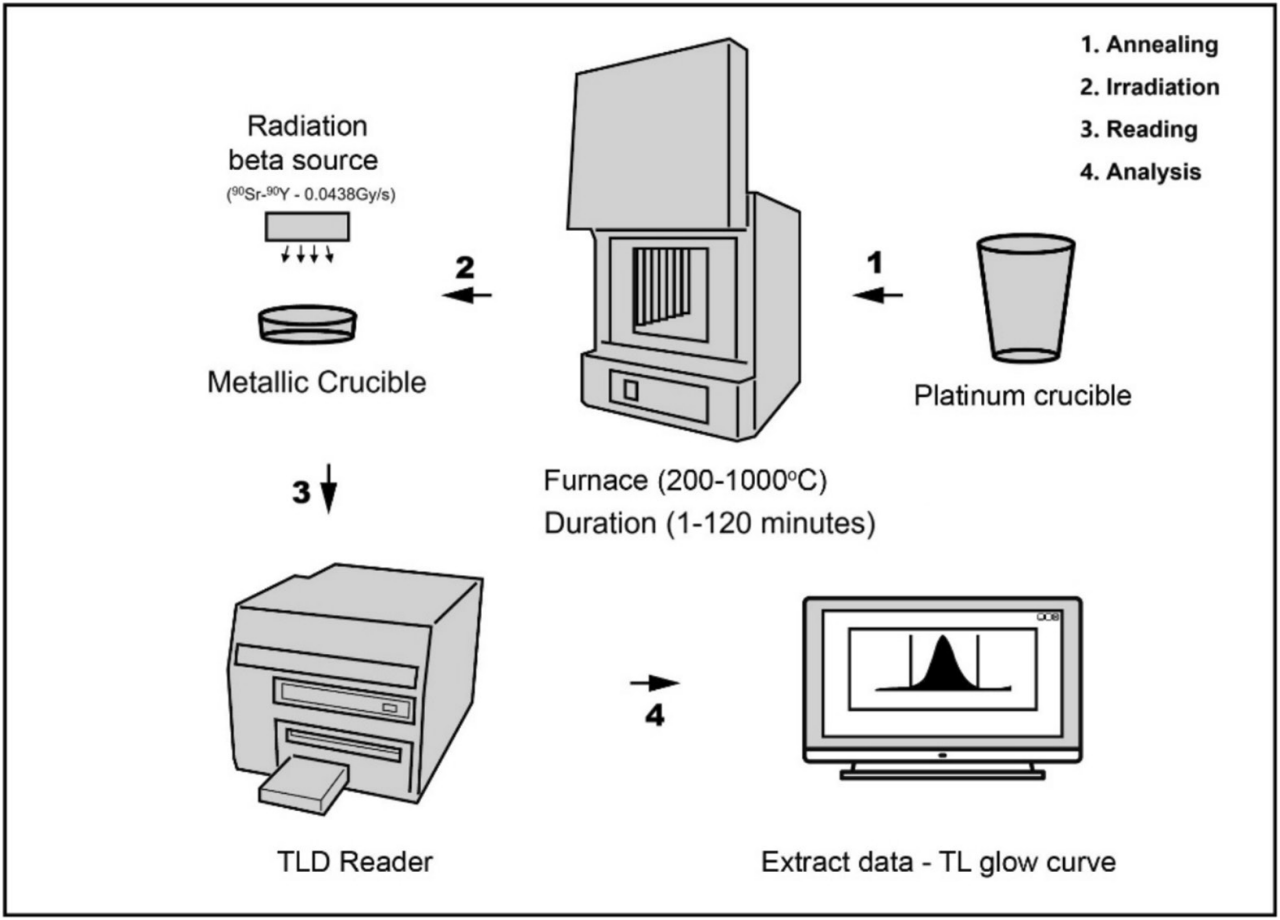
Schematic representation of the experimental procedure: The annealing process (200°C–1000°C, 1–120 min) for 25 mg of h‐BN powder samples irradiated with a β‐source (^90^Sr–^90^Y, 0.04386 Gy/s) in platinum and metallic crucibles. After annealing, the samples are heated in a TLD reader, which transmits the TL glow curves data to a connected computer.

Two experiments were conducted in this study to evaluate the effects of different annealing processes on the TL behavior of h‐BN samples. The first experiment investigated the influence of varying annealing temperatures, ranging from 200°C to 1000°C, with a constant annealing duration of 30 min for nine 25 mg h‐BN powder samples. The second experiment focused on the effects of different annealing times of 1, 5, 15, 30, 60, and 120 min—at a constant temperature of 900°C, using six 25 mg h‐BN samples. All annealing processes were performed in air atmosphere. Every annealed sample was irradiated with 72 Gy, and then they were read in the TLD reader starting at 30°C and finishing at 400°C, with a heating rate of 1°C/s. The experimental procedures are explained figuratively in Figure 4. All aliquots were read out before each irradiation procedure to evacuate the traps and leave them empty of all residual natural dose and to determine the background dose and subtract it from the main TL measurement.

## 4 Results

### 4.1 SEM and XRD

According to CY Engineering, XRD analyses for the original samples (non‐annealed) were performed with the Hueber G670 transmission detector, copper generator, and standard 15‐min measurements between 5° and 110° of 2‐Theta values as shown in Figure 6a. The XRD analysis for annealed samples in Figure 6b,c was implemented at Middle East Technical University, the chemistry department laboratory in Ankara, Turkey. Comparing XRD results for annealed and non‐annealed samples confirmed the same crystal pattern and h‐BN crystal dimensions.

**FIGURE 5:**
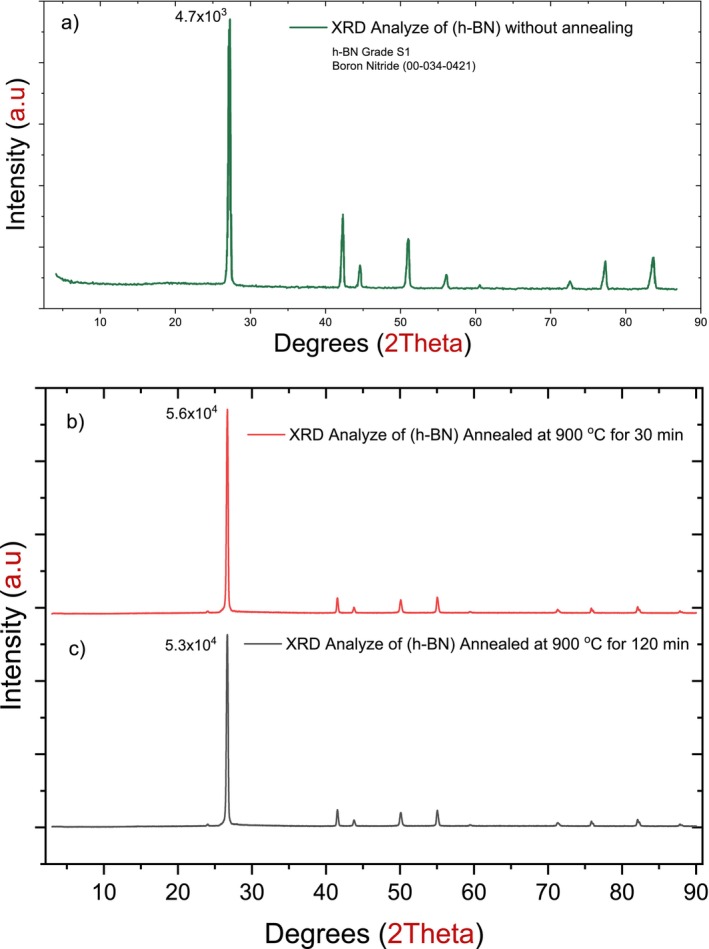
The XRD analyses of hexagonal boron nitride (h‐BN): a) as received according to the CY Lab, b) annealed at 900°C for 30 min, and c) annealed at 900°C for 120 min.

**FIGURE 6:**
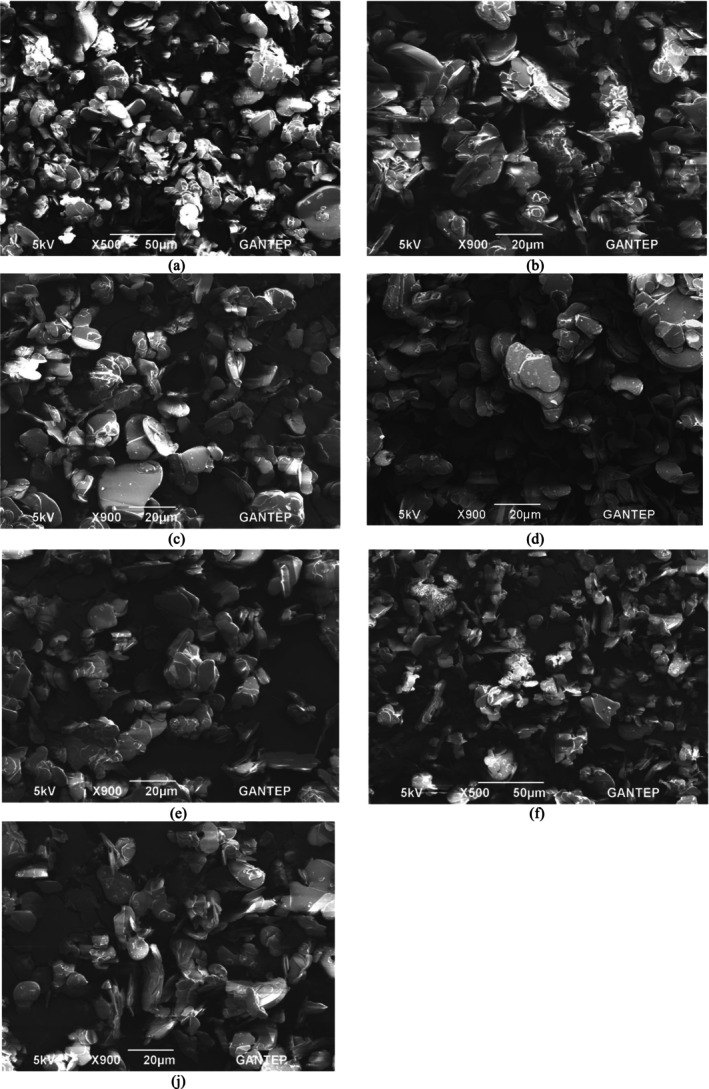
SEM images of h‐BN micropowder under various annealing process and magnifications: a) without annealing, 50 μm; b) without annealing, 20 μm; c) annealed at 300°C for 30 min, 20 μm; d) annealed at 400°C for 30 min, 20 μm; e) annealed at 900°C for 30 min, 20 μm; f) annealed at 1000°C for 30 min, 50 μm; j) annealed at 1000°C for 30 min, 20 μm.

The position (2θ) of the main peak in the XRD patterns was proximate, as shown in Table 2 for annealed and non‐annealed samples; also, the orientation of the crystal plane for h‐BN described by the Miller Indices (002) was the same [20]. However, higher annealing temperature provides the atoms within the h‐BN crystal with high energy resulting in enhancement of the mobility and decrease of the defects, which can be concluded from the increased intensity [21], as seen in Figure 5b,c and scanning electron microscopy (SEM) analysis (Figure 6d). The SEM photomicrographs of the samples were taken using the ZEISS Gemini SEM 300 device at the Central Laboratory of Gaziantep University.

**TABLE 2:** The main peak ID extended report.

h k l	2θ (degrees)	*d* (A^o^)	Height (%)	Sample type
0 0 2	~27	3.299	46	Without annealing
0 0 2	26.69	3.337	561	Annealed at 900°C for 30 min
0 0 2	26.66	3.341	527	Annealed at 900°C for 120 min

Figure 6a,b shows the SEM analysis of the original h‐BN sample (without annealing) at magnifications of 50 and 20 μm, respectively; Figure 6c–e corresponds to samples annealed at 300°C, 400°C, and 900°C for 30 min, all observed at 20 μm. Figure 6f presents the sample annealed at 1000°C for 30 min at 50 μm magnification, while Figure 6j shows the same condition captured at 20 μm.

The surface of the original sample exhibits a rough and irregular texture with various particle shapes and sizes, indicating a polycrystalline structure or a non‐uniform surface. In contrast, the annealed samples exhibit a smoother appearance in some areas.

In the annealed samples, a noticeable change in particle structure enhanced the crystallinity of the h‐BN and led to a more stable and uniform microstructure, which was evidenced by higher intensity in XRD [22]. This includes grain growth, particle merging, larger and more defined grains, and a reduction in smaller, fragmented particles; internal stresses and defects were also observed.

### 4.2 Applying Various Annealing Temperatures at Constant Time

**FIGURE 7:**
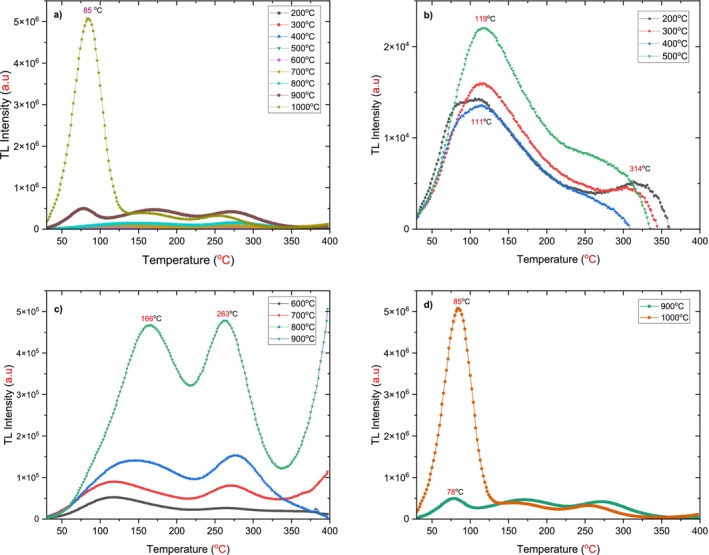
Thermoluminescence (TL) glow curves of h‐BN measured at various annealing temperatures for 30 min, with a heating rate *β* = 1 following 72 Gy beta irradiation.a) Total range (200°C–1000°C); b) low‐temperature range (200°C–500°C), showing a prominent peak at 119°C and a minor peak at 314°C; c) high‐temperature range (600°C–900°C), exhibiting two dosimetric peaks at 166°C and 263°C; d) shallow‐temperature range (900°C–1000°C), dominated by a shallow peak at 85°C.

In this technique, different annealing temperatures, ranging from 200°C to 1000°C, were applied at a constant annealing time of 30 min to nine h‐BN powder samples of 25 mg. The samples were then irradiated with a dose of 72 Gy. Finally, after 10 min in the darkroom, the irradiated samples were read out using a TLD reader, starting at 30°C and finishing at 400°C, with a heating rate of 1°C/s. In this experiment, we study the effect of different annealing temperatures at a constant time on the behavior of the TL glow curve of h‐BN, as shown in Figure 7a–d.

Figure 7a shows the TL glow curve of h‐BN for various annealing temperatures ranging from 200°C to 1000°C at a constant annealing time of 30 min. The TL glow curve depicts a shallow peak around 85°C, which begins to form at 900°C and becomes sharp and dominant at 1000°C, with a significant increase in intensity. To gain a deeper understanding of the influence of annealing on TL behavior, the resultant TL glow curves should be analyzed across all applicable temperatures. This can be achieved by categorizing them into three distinct regions based on the applied temperatures.

The first region corresponds to the low annealing temperature range, applied between 200°C and 500°C. Figure 7b shows the resulting TL glow curves, where two peaks were observed: the first peak was located around 111°C, while the second peak, a small and broad peak attributed to overlapping peaks or defect states, appeared around 314°C [12, 19]. Its intensity was approximately 2.8 times lower than the first peak that emerged at 200°C. At 300°C, the first peak became more distinct and shifted to 117°C, while the deeper peak was observed around 304°C; however, no significant change in intensity was noted. At higher temperatures, up to 500°C, the first peak shifted further into the deeper peak region by 8°C, reaching 119°C. In contrast, the second peak still exhibited a continuous shape and was small.

**FIGURE 8:**
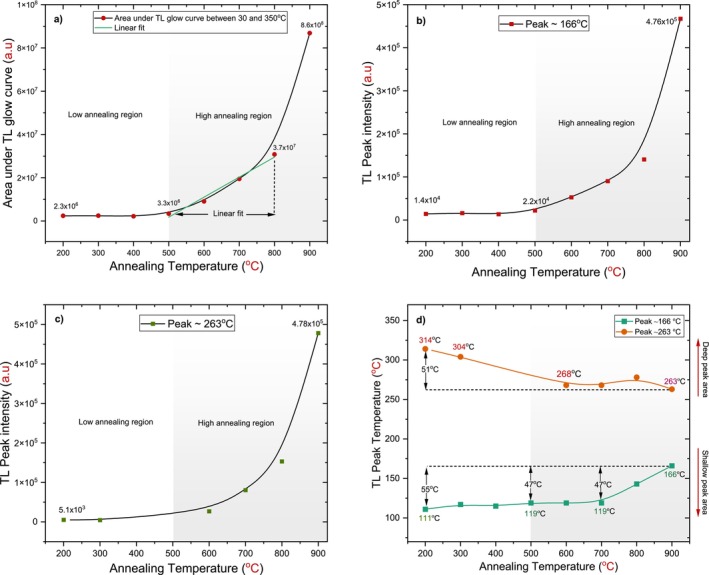
Variations as a function of annealing temperature for the h‐BN samples.a) The area under the TL glow curve between 30°C and 350°C. b) The TL intensity of the peak at 166°C. c) The TL intensity of the peak at 263°C. d) The peak temperatures at 166°C and 263°C.

The total number of trapped charge carriers—determined by the area under the TL glow curve—as well as the maximum intensity of emitted lights, increased slowly with higher annealing temperatures in this region, as shown in Figure 8a–c. This suggests a potentially limited influence of low temperature annealing on the TL behavior of h‐BN.

In the higher annealing temperature region of 600°C to 900°C, two promising dosimetric peaks were identified, located around 166°C and 263°C, as shown in Figure 7c. The first peak, which initially appeared in the lower‐temperature region, maintained its position of 119°C for annealing temperatures of 600°C and 700°C. It shifted to a deeper region by 47°C at both 800°C and 900°C, finally stabilizing around 166°C, as illustrated in Figure 8d, which explains the variations of the peak temperatures as a function of annealing temperature. A second peak, observed in the deeper region, was initially located around 268°C for the 600°C and 700°C annealing temperatures. At 900°C, this peak shifted slightly toward a shallower region by 5°C, ultimately stabilized around 263°C. Notably, the shape of these peaks became more distinct, and the TL glow curve conformed to the ideal characteristics required for dosimetry studies in alignment with TL theory [18, 19]. The observed increases of the TL between annealing temperatures of 500°C and 800°C highlight the significant role of the annealing, as illustrated in Figure 8a–c and Table 3.

**TABLE 3:** The maximum intensity for peaks around 166°C and 263°C and the area under the TL glow curves between 30 and 350°C for annealing degrees between 200°C and 900°C.

Annealing temperature (°C)	Intensity of ~166°C (a.u.) × 10^4^	Intensity of ~263°C (a.u.) × 10^4^	The area between 30°C and 350°C (a.u.) × 10^6^
200	1.42	0.51	2.39
300	1.59	0.45	2.45
400	1.35	—	2.18
500	2.20	—	3.38
600	5.26	2.67	9.06
700	9.02	8.05	19.43
800	14.06	15.30	30.78
900	46.73	47.81	86.86

At a higher annealing temperature of 900°C in the third region, the shallow peak located around 78°C transformed to locate around 85°C at 1000°C as in Figure 7d; the TL intensity and area of the shallow peak increased dramatically; its intensity was much greater at 1000°C compared to its value recorded at 900°C. The other two peaks diminished and could be neglected for the highest degree applied.

**FIGURE 9:**
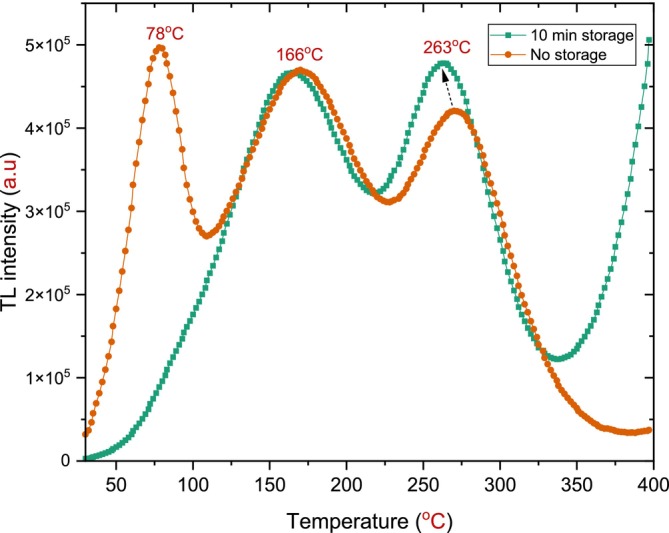
The difference between the faded (10 min in a dark room) and non‐waited TL glow curve for h‐BN annealed at 900°C for a constant time of 30 min with *β* = 1°C/s after beta irradiation of 72 Gy.

It is important to note that, originally, the TL glow curve obtained at 900°C included a shallow peak located around 78°C. In shallow peaks, small excitons in the lattice make trapping and recombining the electron–hole pairs easy and rapid, which is not desired in TL dosimeter studies [23]. These transitions can occur at room temperature, as evidenced by the results of this study. Specifically, the shallow peak completely vanished after 10 min of storage in a dark room following the irradiation process. Figure 9 illustrates the difference between the TL glow curve after 10 min of fading and the non‐waited TL glow curves for the same h‐BN sample subjected to the same annealing process (900°C for 30 min).

This experiment was repeated twice to confirm that the shallow peak at approximately 78°C completely faded after 10 min of storage, where the intensity significantly reduced and the peak emptied of electrons; at room temperature, trapped electrons in the shallow peak were released and either recombined at the luminescence center or were re‐trapped in deeper traps, which explains the observed increase in the intensity of the second dosimetric peak [20].

### 4.3 Applying Different Annealing Times at Constant Temperature

**FIGURE 10:**
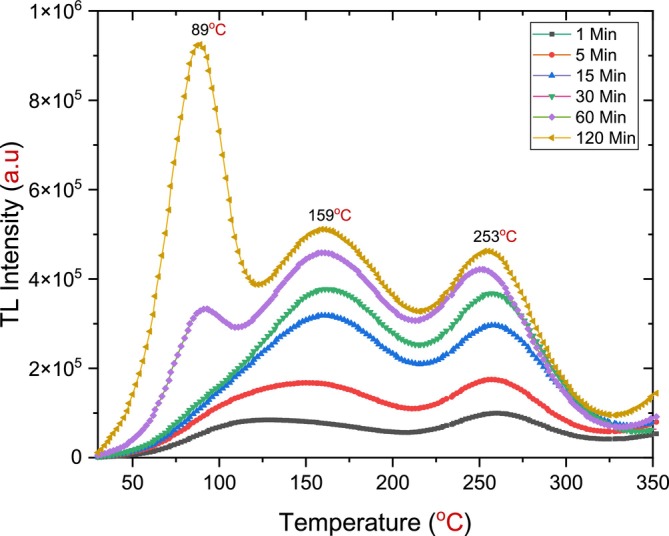
A TL glow curve of hexagonal boron nitride (h‐BN) annealed at different times at a constant temperature of 900°C with *β* = 1°C/s after beta irradiation of 72 Gy.

Since a good TL glow curve was obtained by applying an annealing temperature of 900°C, it was selected for further experimentation. This study used different annealing durations (from 1 to 120 min) for 25 mg of h‐BN powder. The samples were then irradiated with a dose of 72 Gy. After 10 min of storage in a dark room, the irradiated samples were read using a TLD reader, starting at 30°C and reaching 400°C, with a heating rate of 1°C/s. The effect of annealing time on the behavior of the TL response of h‐BN, as shown in Figure 10, was studied and analyzed.

A good TL glow curve was obtained, as shown in Figure 10; this curve featured two peaks at approximately 161°C and 257°C. Both peaks began to form at an annealing time of 1 min as a broad peak. However, with the increasing annealing time to 30 min, the peaks became sharper and more distinct as individual peaks, enabling the determination of kinetic parameters. [18, 19].

In addition to these dosimetric peaks, a shallow peak was observed at high annealing times (60 and 120 min) located around 89°C. This peak began to form at an annealing time of 60 min at approximately 93°C and shifted to 89°C as the annealing time increased to 120 min. This peak emerged despite the procedure implemented in the first technique to prevent such results: storing the irradiated sample in a dark room for 10 min before reading it in the TLD reader.

**FIGURE 11:**
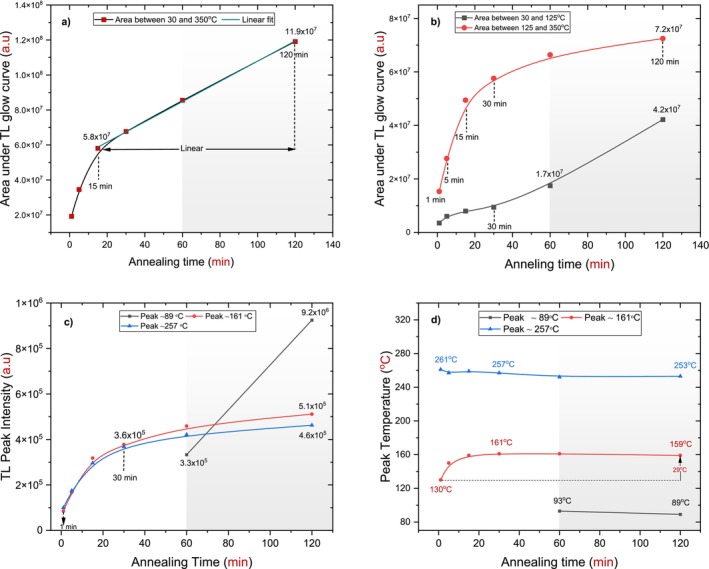
Variations as a function of annealing time (1–120 min) for the h‐BN sample. a) The area TL glow curve between 30°C and 350°C, with a linear fit applied to the range of 15–120 min. b) The partial areas under the TL glow curve divided into two regions: 30°C–125°C and 125°C–350°C. c) The TL intensity for the peaks at 89°C, 161°C, and 257°C. d) The peak temperatures at 89°C, 161°C, and 257°C.

Across all annealing times, a slight shift in the peak located around 257°C was observed, moving toward the shallower peak region by approximately 8°C; at 120 min, it transformed to around 253°C. In contrast, the first peak shifted toward the deeper peak area at a higher annealing time applied by approximately 29°C; at 120 min, it was located around 159°C as shown in Figure 11.

The area under the TL glow curve between 30°C and 350°C was investigated as a function of annealing time, ranging from 1 to 120 min. The concentration of trapped charge carriers (electron–hole pairs) increased with annealing time. A well‐defined linear fit was observed for annealing times of 15, 30, 60, and 120 min, as shown in Figure 11a. This linear fit could not be accurate due to the intervention of a shallow peak, particularly at higher annealing times. Given the emergence of these shallow peaks, it is useful to divide the area into two parts. This approach helps identify the increasing pattern of trapped charge carriers under the dosimetric peaks around 161°C and 257°C, thereby highlighting the influence of annealing time [24].

The first part of the area under the TL glow curve extended from 30°C to 125°C. This range was chosen because it corresponds to a shallow peak area where the effect of annealing time is generally evident. A significant increase was observed at 60 and 120 min, attributed to the appearance of shallow peaks around 93°C and 89°C, respectively. The second part of the area, ranging from 125°C to 350°C, was selected as it encompasses the dosimetric peaks. A dramatic increase was noted for annealing times of 1, 5, and 15 min, followed by a slightly elevated response at 30, 60, and 120 min, as shown in Figure 11b.

Figure 11c shows the same behavior for TL peak intensities, but it was different for the shallow peak, as it increased dramatically between 60 and 120 min. In each step of the annealing time, approximate intensity values for the dosimetric peaks were recorded, as shown in Table 4.

**TABLE 4:** Intensities for peaks located around 161 and 257°C for annealed h‐BN at a constant temperature of 900°C at different times ranging from 5 to 120 min.

Annealing time (min)	Intensity of ~161°C (a.u.) × 10^5^	Intensity of ~257°C (a.u.) × 10^5^
5	1.6	1.7
15	3	2.9
30	3.7	3.6
60	4.5	4.2
120	5	4.6

The first peak began to form at around 130°C as a continuous peak. With an increasing annealing duration, it transformed into a distinct peak at the final applied duration of 120 min, centering at 159°C. It shifted by 29°C toward deeper peaks throughout the six annealing stages. In contrast, the second peak initially appeared at 261°C and stabilized around 253°C at the final annealing duration. The shift in its position was minimal and negligible, as seen in Figure 11d.

Finally, the TL glow curve exhibited similar behavior across different annealing temperatures and durations. The same glow curve pattern was observed, featuring two dosimetric peaks: the first located between 161°C and 166°C and the second between 257°C and 263°C. When comparing peak intensities at an annealing temperature of 900°C for 60 and 120 min, the increase in intensity was minimal, suggesting that the TL behavior remains consistent with extended annealing durations. Although all irradiated samples were stored in a dark room for 10 min to mitigate the impact of shallow peaks, these peaks continued to appear distinctly at longer annealing durations (60 and 120 min), as shown in Figure 10, and at higher annealing temperatures (1000°C), as illustrated in Figure 7d.

## 5 Discussion

This research analyzed the effect of annealing processes, represented by temperature and time, on the TL characteristics of the wide bandgap semiconductor and Group III–nitride h‐BN. The first process was investigated by applying nine different temperature levels ranging from 200°C to 1000°C at a constant duration of 30 min. The second process was applying multiple durations ranging from 1 to 120 min at a constant temperature of 900°C.

The results indicate three patterns of TL glow curves: the first is obtained at low annealing temperatures (200°C–500°C at 30 min), where the glow curve has mainly one peak located around 119°C and a small broad deep peak around 314°C. The second is obtained at high annealing temperatures (600°C–900°C at 30 min) and at annealing time (1–30 min at 900°C), where two promising peaks are found in the glow curves; they are located around 161°C°C–166°C and 257°C–263°C. The first peak is linked to hydrogenated boron vacancies, carbon substitutional defects, or oxygen substitutional impurities. These centers form during annealing in air and are energetically stable enough to trap carriers until released at moderate temperatures. Previous studies on h‐BN confirm that such defect types produce mid‐gap levels and play active roles in both TL and PL behavior [16, 25], while the second peak in the pattern is associated with stable intrinsic defects such as isolated boron vacancies, oxygen substitution complexes, or vacancy–impurity complexes. These defects introduce deep‐level traps requiring higher thermal energy to release carriers. They are particularly important in dosimetric applications due to their strong thermal stability and persistent trapping behavior [25, 26]. These defect assignments are consistent with the behavior of TL glow curves under varying annealing temperatures and times. As the annealing process increases charge carriers mobility in the crystal and promotes impurity diffusion (especially oxygen in air), these defects become thermally activated, enabling the formation of clearly resolved TL peaks. The results therefore confirm that defect engineering through annealing is a critical mechanism in defining the TL response of h‐BN.

The third pattern resulted from a high annealing process (1000°C for 30 min and 900°C for 60 and 120 min); the integrated glow curve exhibits an additional shallow peak around 85°C–89°C. This peak can be attributed to surface states, grain boundary defects, and weakly adsorbed oxygen species introduced at high annealing temperatures (≥900°C) [23, 26, 27]. As temperature and time increase, the area and TL peak intensity increase in all stages, which can be attributed to the annealing effects illustrated in several studies [24, 28, 29, 30].

The second pattern provides important indicators of the prominent effects of annealing on TL behavior, as the resulting TL glow curves resemble and feature two peaks located around 160°C and 255°C. The resultant plots in this pattern are preferable for TL characteristics according to the theory of TL and related phenomena [18, 19]. At each stage, both peaks showed similar intensity values and areas under the peaks. Furthermore, the two peaks exhibited comparable behavior regarding their locations. When the first factor of the annealing process changed between 600°C and 900°C, the first peak shifted deeper by 31°C, eventually positioning around 166°C, while the second peak shifted shallower by 5°C, ultimately locating at 263°C. When the second factor varied between 1 and 30 min, the first peak moved deeper by 31°C, centering around 161°C, while the second became shallower by 4°C, locating around 253°C. This behavior can be associated with the influence of annealing; as annealing increases, some peaks shift to deeper regions, indicating an increase in trap depth. This behavior is considered somewhat rare, as most studies on the effect of thermal treatment report a decrease in trap depth [24, 28].

**FIGURE 12:**
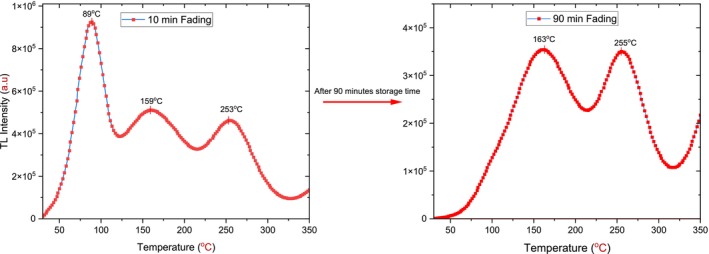
The TL glow curves of annealed h‐BN at 900°C for 120 min after 10 and 90 min of storage time.

However, capturing free charge carriers in deep peaks and preventing recombination are essential to studying the TL characteristics of the materials. More precisely, the confinement of free electrons for longer durations leads to a good understanding of semiconductors' optical properties [31]. Yet, the appearance of shallow peaks leads to negative results; the trapped charge carriers in shallow peaks release and recombine rapidly, decreasing TL peak intensity and reducing sensitivity, leading to a nonideal TL signal and less efficiency [27, 32]. To surpass the impact of the shallow peak, the TL glow curve resulting from annealing at 900°C for 120 min, and featuring three peaks around 89°C, 159°C, and 253°C, was transformed into a second (two‐peak) pattern after 90 min of storage time, as shown in Figure 12.

The improved microstructural properties observed in XRD and SEM integrated with the high annealing processes culminated in a significant increase in the TL by excitonic transitions. This enhancement in TL is a consequence of the improved crystallinity, stability, and reduced structural disorder in the h‐BN.

## 6 Conclusion

**FIGURE 13:**
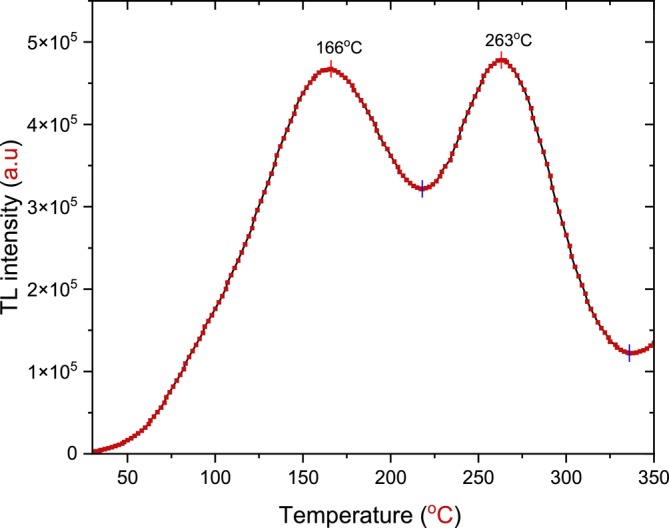
A TL glow curve of wide bandgap semiconductor hexagonal boron nitride (WBG/SC‐h‐BN) annealed at constant time and temperature (900°C, 30 min) stored in a dark room for 10 min, with *β* = 1°C/s after beta irradiation of 72 Gy.

In this study, two experiments were conducted to determine the typical annealing processes by analyzing the results, comparing the TL glow curves. To save time, money, and effort, we conclude that the ideal and effective annealing process is a temperature of 900°C and a time of 30 min, as well as 10 min of storage time. This demonstrates good behavior of the TL properties for h‐BN; the resulting TL glow curve is shown in Figure 13. To further assess the impact of annealing on crystallinity and structural properties, it is recommended to conduct additional analyses such as Raman spectroscopy and transmission electron microscopy. The appearance of shallow peaks is considered a negative outcome in this study, which calls for further investigations to identify their causes and explore effective ways to mitigate their impacts.

## Conflicts of Interest

The authors declare no conflicts of interest.

## Data Availability

The data that support the findings of this study are available from the corresponding author upon reasonable request.
